# From photosensitivity to autoimmunity: the role of AI metabolomics in PCT–celiac disease overlap

**DOI:** 10.1097/MS9.0000000000004730

**Published:** 2026-01-14

**Authors:** S. Tongpangkokla Ozukum, Kainat Wajahat, Muhammad Abdullah, Maliha Khalid, Aminath Waafira

**Affiliations:** a Department of Medicine, IMDH District Hospital College of Nursing, Nagaland; b Department of Medicine, Liaquat University of Medical and Health Sciences (LUMHS), Karachi, Pakistan; c Shaheed Zulfiqar Ali Bhutto Medical University, Karachi, Pakistan; d Department of Medicine, Jinnah Sindh Medical University, Karachi, Pakistan; e School of Medicine, The Maldives National University, Malé, Maldives

**Keywords:** artificial intelligence, celiac disease, metabolomics, porphyria cutanea tarda

## Abstract

Porphyria cutanea tarda (PCT), caused by hepatic uroporphyrinogen decarboxylase deficiency, may rarely overlap with undiagnosed celiac disease (CD), complicating diagnosis due to shared hepatic and dermatologic features. Both conditions present with skin manifestations such as photosensitivity, blistering, or dermatitis herpetiformis-like lesions, often leading to misclassification. AI-driven metabolomics provides a novel strategy to identify metabolic biomarkers, predict CD with high accuracy through machine learning, and uncover hidden comorbidities in PCT patients. Despite challenges including limited datasets, bioinformatics demands, and regulatory barriers, integrating metabolomics with AI could enhance diagnostic precision and enable personalized management. Multidisciplinary collaboration is critical to translating these technologies into clinical practice for early detection and improved outcomes in PCT–CD overlap.

*To the Editor,*

Porphyria cutanea tarda (PCT) arises from hepatic uroporphyrinogen decarboxylase deficiency. It leads to porphyrin accumulation and skin-liver manifestations. A rare association with undiagnosed celiac disease (CD) is often overlooked. CD is an autoimmune disorder with atypical hepatic or dermatological signs^[1,2]^. This overlap can delay diagnosis and hinder management. AI-driven metabolomics can uncover hidden comorbidities via metabolic biomarkers. Integrating these tools may enable earlier detection and personalized treatment.

CD is predicted using AI and an autoimmune gene panel. Various techniques like logistic regression, SVM, and neural networks were utilized. Machine learning predicts CD with high accuracy. PCT is identified via laboratory methods. High porphyrin levels in urine, blood, and stool indicate PCT^[3]^. AI-driven modeling enables CD prediction. Combining methods aids in diagnosis and management.

Porphyrias were sometimes accidentally diagnosed with CD. Managing patients with both CD and porphyria poses challenges. Porphyrias are rare illnesses affecting heme biosynthesis, causing porphyrin build-up. This build-up leads to skin issues like pigmentation, bullae, and scarring in sun-exposed areas. CD is linked to dermatitis herpetiformis, which is similar to porphyria skin lesions. Variegate porphyria involves the buildup of porphobilinogen and 5-aminolevulinic acid. This leads to acute neuropsychiatric and gastrointestinal symptoms; certain drugs like dapsone can trigger episodes^[4]^.

AI metabolomics has clear advantages but faces significant issues in healthcare adoption. Issues include the need for bioinformatics infrastructure, high costs, and high-precision data needs. Training AI models requires many samples, which presents a significant challenge^[5]^. Limited clinical data for rare diseases makes building reliable models difficult. The broad applicability of results is unclear. Healthcare use is limited by challenges in interpreting algorithm outputs and regulatory standards. Unintentional negative health consequences of new algorithms are a concern. Overcoming challenges requires cooperation from fields like dermatology, pathology, data science, and gastroenterology^[6]^.

In conclusion, PCT and CD may share a metabolic link. AI and machine learning advancements enable high-accuracy CD prediction. Metabolomics helps detect biochemical markers of both diseases. Similar symptoms, like photosensitive rashes, can lead to misdiagnosis. AI-integrated metabolomic diagnostics aid in early detection and management. Multidisciplinary collaborations are essential for the clinical implementation of these tools. This letter to the editor is in compliance with the TITAN guideline^[7]^.

## Data Availability

No data were generated for this manuscript.

## Research registration unique identifying number (UIN)

(1) Name of the registry: N/A. (2) Unique Identifying number or registration ID: N/A. (3) Hyperlink to your specific registration (must be publicly accessible and will be checked): N/A.
